# Efficacy and Safety of Ketone Supplementation or Ketogenic Diets for Alzheimer's Disease: A Mini Review

**DOI:** 10.3389/fnut.2021.807970

**Published:** 2022-01-17

**Authors:** Matthieu Lilamand, François Mouton-Liger, Emmanuelle Di Valentin, Marta Sànchez Ortiz, Claire Paquet

**Affiliations:** ^1^Groupe Hospitalier Lariboisiere FW Saint-Louis, Cognitive Neurology Center Paris Nord Ile de France, AP-HP.Nord, Paris, France; ^2^Department of Geriatrics, Bichat and Bretonneau University Hospitals, AP-HP.Nord, Paris, France; ^3^INSERM UMR-S1144, Université de Paris, Paris, France; ^4^Department of Histology and Biology of Aging, Groupe Hospitalier Lariboisiere FW Saint Louis, AP-HP.Nord, Paris, France

**Keywords:** Alzheimer's disease, nutrition, ketone bodies, ketogenic diet, medium chain triglycerides

## Abstract

Alzheimer's disease (AD) is the most frequent age-related neurodegenerative disorder, with no curative treatment available so far. Alongside the brain deposition of β-amyloid peptide and hyperphosphorylated tau, neuroinflammation triggered by the innate immune response in the central nervous system, plays a central role in the pathogenesis of AD. Glucose usually represents the main fuel for the brain. Glucose metabolism has been related to neuroinflammation, but also with AD lesions. Hyperglycemia promotes oxidative stress and neurodegeneration. Insulinoresistance (e.g., in type 2 diabetes) or low IGF-1 levels are associated with increased β-amyloid production. However, in the absence of glucose, the brain may use another fuel: ketone bodies (KB) produced by oxidation of fatty acids. Over the last decade, ketogenic interventions i.e., ketogenic diets (KD) with very low carbohydrate intake or ketogenic supplementation (KS) based on medium-chain triglycerides (MCT) consumption, have been studied in AD animal models, as well as in AD patients. These interventional studies reported interesting clinical improvements in animals and decrease in neuroinflammation, β-amyloid and tau accumulation. In clinical studies, KS and KD were associated with better cognition, but also improved brain metabolism and AD biomarkers. This review summarizes the available evidence regarding KS/KD as therapeutic options for individuals with AD. We also discuss the current issues and potential adverse effects associated with these nutritional interventions. Finally, we propose an overview of ongoing and future registered trials in this promising field.

## Introduction

Alzheimer's disease (AD) is the most frequent age-related neurodegenerative disorder, characterized by brain abnormal deposition of β-amyloid peptide (Aβ) and phosphorylated tau (pTau) accumulation. These protein deposits cause neuronal death, cognitive decline and behavior disorders (1). Industrial research has focused on disease-modifying therapeutics (mostly anti-amyloid agents), which have failed so far to significantly change the course of the disease (2). Nutrition has been suggested as a key modifiable factor to fight against protein-driven cognitive symptoms or to postpone the onset of cognitive impairment (3). Aβ deposition and tau lesions are accompanied by activated microglia and astrocytes (4). These cells release proinflammatory cytokines and chemokines causing chronic neuroinflammation. In turn, neuroinflammation promotes neurodegeneration and Aβ production. Hyperglycemia is also associated with neuroinflammation, leading to oxidative stress which could trigger the amyloid cascade of AD. Several other pathophysiological links exist between AD and glucose metabolism. In cognitively normal older adults, a high-glycemic diet was previously shown to be associated with cerebral Aβ burden (5). Very recently, in a longitudinal analysis over one year, the same research team found greater brain Aβ accumulation in participants with higher daily intake of sugar or carbohydrates (6). AD my also result from insulin resistance, which affects insulin signaling and favors the deposition of brain Aβ and pTau (7). Besides, type 2 diabetes which is an established risk factor for AD, shares in common the deleterious consequences of the expression of *apolipoprotein E allele 4* (*ApoE4*) (8). Carrying the *ApoE4* allele represents the main genetic risk factor for sporadic AD, indicating a likely common pathophysiological background with diabetes. As demonstrated by fluorodeoxyglucose positron emission tomography (FDG-PET), abnormal brain glucose metabolism in the temporal and parietal lobes occurs from the earliest stages, in AD animal models and AD patients, but also in asymptomatic individuals at risk of AD. Interestingly, these hypometabolic brain areas demonstrate impaired glucose utilization, whereas they may still uptake ketone bodies efficiently (KB) (9).

These findings have shed the light on the potential therapeutic use of KB in the field of AD therapeutic research. Two different strategies have been considered: first, the intake of medium-chain triglycerides (MCT) resulting in the production of KB after beta-oxidation in the liver. Thus, KB cross the blood brain barrier and fuel the brain. Second, when glucose is not readily available (e.g., starvation), a metabolic switch occurs in favor of KB usually released by the liver. The diets specifically designed for KB production are called ketogenic diets (KD) (10). The core characteristics of the KD are the association of a high amount of fat, with low carbohydrate intake: usually a macronutrient ratio of fat to protein and carbohydrate combined equal to 3 or 4:1. When ketosis is achieved, the main fuel used by the body shifts from glucose to favor KB, an adaptation that also occurs with extended fasting.

In this short review, we examined the current very recent (since May 2019) available evidence regarding KS/KD as therapeutic options for AD. As these nutritional interventions may raise major concerns in older adults or frail individuals suffering from AD, we also discuss the controversies and potential adverse effects due to KS or KD. Finally, we propose an overview of ongoing and future registered trials, in this promising field.

## Recent Findings and Research Gaps

As highlighted in previously published reviews in this field (11, 12), KD or KS in animal models have repeatedly shown improvements of cognitive performance, motor function or behavior. These clinical benefits were accompanied with decreased inflammatory markers, but also with alleviated brain Aβ load or tau (neurofibrillary tangles) burden. In humans, a growing body of evidence has also suggested the potential interest of KS or KD to slow down cognitive decline or to enhance cognition. Several studies have pointed out the change in brain metabolism assessed by PET imaging, after KS.

Since 2019, we found ten studies, in humans, aiming at improving cognitive performance or biomarkers of AD (See Table 1) (13–22). Among them, seven used KS vs. placebo and three KD vs. control diet. Of note, the most valuable clinical investigation regarding KS included 413 older participants (mean age = 77) with mild to moderate AD, followed-up over 6 months (15). The intervention consisted in 20 g of MCT supplementation, a standard daily dose to produce KB. Finally, Henderson et al. did not observe significant improvements regarding their two main endpoints i.e., cognitive performance and clinician's impression of change. On the other hand, in older adults with mild cognitive impairment (MCI) Fortier et al. showed that 30 g of MCT supplementation over a 6-month follow, significantly improved three major cognitive functions: episodic memory, executive function and language (19). Three studies in healthy participants used KS over very short intervention windows (0–5weeks) and reported improved cognitive functions and/or brain metabolism (14, 16, 22).

**Table 1:** Studies of ketone supplementation or ketogenic diet with regards to cognition or AD biomarkers.

**Study (ref)**	**Population**	**Mean age (SD)**	**Intervention**	**N**	**Follow-up (weeks)**	**Outcomes**	**Positive outcomes (intervention group)**	**Nutritional changes**	**Side effects**
Philipps et al. (13)	Mild AD	70	RCT crossover, KD vs. CD	26	12 × 2	Cognition, IADL, QoL	Improved IADL performance and QoL	Weight loss	Mild. One (4%) withdrawal due to KD
Yomogida et al. (14)	Healthy adults	66	RCT crossover, MCT (50g vs. placebo)	20	–	Cognition, fMRI	Improved executive function and brain metabolism	Unassessed	Unassessed
Henderson et al. (15)	Mild to moderate AD	77	RCT, MCT 20g vs. placebo	413	26	Cognition, clinician's impression of change (ADCS-CGIC)	No cognitive change	No nutritional disorder reported	Minor digestive side effects
O'Neill et al. (16)	Healthy adults	60	RCT crossover, MCT (30 g) vs. placebo	80	2	Cognition, adverse effects	No cognitive change	Unassessed	Minor digestive side effects
Nagpal et al. (17)	Adults with MCI/CN	65	RCT crossover, KD vs. CD	17	6 × 2	CSF biomarkers, microbiome	CSF biomarkers improvement	Unassessed	Unassessed
Neth et al. (18)	Adults with SCI/MCI	64	RCT, KD vs. CD	20	6 × 2	Cognition, PET imaging, CSF biomarkers	Improved memory, brain metabolism and CSF biomarkers	Weight loss	None
Fortier et al. (19)	Adults with MCI	72	RCT MCT (30 g) vs. placebo	122	26	cognition	Improved cognition memory, executive function, language	Stable weight	Minor digestive adverse effects
Xu et al. (20)	Mild moderate AD	75	RCT crossover, MCT (17 g)-leucin vs. placebo	46	4 × 2	cognition	Improved cognition	Unassessed	None
Abe et al. (21)	Nursing home residents	85	RCT, MCT (6 g)-leucin vs. placebo	64	13	cognition	Improved cognition	Unassessed	None
Ashton et al. (22)	Healthy students	20	RCT, MCT (12 or 18 g) vs. placebo	30	5	cognition	Improved executive function/working memory	Unassessed	Minor digestive side effects

*AD, Alzheimer's disease; ADCS-CGIC, Alzheimer's Disease Cooperative Study—Clinical Global Impression of Change; CD, control diet; GI, gastro-intestinal; KB, ketone bodies; KD, ketogenic diet; KS, ketone supplementation; MCI, mild cognitive impairment; N, number; PET, positon emission tomography; QoL, quality of life; RCT, randomized controlled trial*.

Three recent studies assessed the effect of a KD on cognition or AD biomarkers (13, 17, 18). In older adults with mild AD, Philipps et al. did not show any significant cognitive gain after 12 weeks of KD vs. CD. However, they reported significant functional improvement in complex activities of daily living, which was considered as clinically meaningful (13). The quality of life of participants under KD also raised of 3 points on the QoL-AD scale. Nagpal et al. and Neth et al. reported significant improvements in memory, brain metabolism and cerebrospinal fluid (CSF) amyloid and tau biomarkers, after only 6 weeks of KD, in adults without dementia (17, 18). The absence of long-term follow-up in these three studies prevented from giving insight into the persistence of cognitive gain (or perception of cognitive improvement) after discontinuation of the nutritional intervention. Adhesion to these nutritional interventions must be carefully examined, since meaningful changes in body composition may occur. More specifically, the monitoring of side effects is crucial in participants with AD, providing that nutritional status is an established predictor of accelerated cognitive or functional decline. In these three trials based on KD two reported weight loss under KD (Nagpal et al. did not follow weight change); in details: Philipps pointed out a mean weight loss of 2.6 kg in the intervention group after only 3 months. This weight loss represents a common adverse consequence of KD, due to drastic restriction in calorie-dense carbohydrates (23).

Several gaps in the available evidence limit our ability to reach a conclusion regarding those interventions in the field of AD. These recent studies display high heterogeneity regarding age, gender ratios or participants' cognitive status, which all influence the risk of subsequent cognitive decline. The limited follow-up durations (<3 months in all studies but two) as well as the repeated cognitive assessments could have induced a retest effect especially in healthy individuals or in adults with early MCI. In addition, the sustainability and the long-term expected benefits (after several years) associated with these interventions remain unknown, so far. Available evidence came from epileptic children monitoring: overall tolerability of KD and efficacy (regarding seizure incidence) were maintained after 6–12 years of treatment (24). Long-term studies are urgently needed in AD patients to assess the feasibility of such diets.

Another major concern for drawing conclusions regarding AD is the definition of the disease. Henderson et al. who included patients with mild to moderate AD, correctly acknowledged that up to 40% of clinically diagnosed (i.e., without CSF or PET biomarkers) patients with AD are likely to be amyloid-negative, hence misdiagnosed (25), which could have resulted in slower progression on cognitive performance under KS. Only two studies of this review (Nagpal, Neth) assessed the change on biological outcomes i.e., CSF biomarkers. Both showed significant improvement after 6 weeks of KD in adults without dementia. The role of *ApoE4* could mitigate the effect of ketone energy metabolism, given that *ApoE4* differentially impacts insulin resistance, glucose metabolism and microglia (26). Yet, KD or MCT intake appeared to be more efficient in non-*ApoE4* carriers (13, 27, 28).

## Ongoing and Future Trials

Among 176 recruiting or active studies related to KD or MCT supplementation, targeting various conditions such as diabetes, epilepsy, glioblastoma, only five were consistent with this topic (see Table 2). The first one (NCT05012748) will describe circulating brain concentrations of free fatty acids under KD, using PET MRI. To be noted, this study will include obese patients between 50 and 70, unlikely to be affected by neurodegenerative conditions. The four other registered trials aim at enrolling AD patients (one of them will also include a subgroup of patients with Parkinson's disease). The kind of nutritional interventions are similar with those described above. Only one future study (NCT04701957) will assess the feasibility of KD in MCI due to AD (confirmed with biomarkers), over 1 year of nutritional follow-up.

**Table 2:** Ongoing trials examining ketone supplementation or ketogenic diet with cognitive outcomes (source: https://clinicaltrials.gov).

**Team**	**CT identifier**	**Enrollment date**	**Design**	**Population**	**Number of participants**	**Follow-up (weeks)**	**Outcomes**
Aarhus University Hospital, Denmark	NCT05012748	Aug 21 → Nov 23	RCT crossover, KD vs. CD	Healthy obese patients 50–70 years	12	2 × 3	Circulating concentrations of free fatty acids (PET MRI)
Cognitive Neurology Center, Assistance Publique Hôpitaux de Paris France	NCT04701957	Feb 21 → Feb 23	RCT, KD vs. CD	Adults with AD (biomarkers)	70	52	Feasibility, cognition, brain metabolism, IADL, side effects
Clinical and Translational Science Unit, University of Kansas Medical Center and NIH, USA	NCT03860792	Oct 19 → Nov 23	RCT, KD + MCT vs. CD	Adults with AD	80	12	Cognition, cerebral concentration of N-Acetylaspartate, blood platelet mitochondrial function
Wake Forest University Health Sciences (USA)	NCT03130036	Jun 15 → Feb 23	Non randomized parallel assignment	Healthy/at risk for AD/early AD	60	–	Brain biodistribution of ketone, [11C]Acetoacetate PET imaging
Université de Sherbrooke, Canada	NCT04322461	Mar 20 → Dec 21	Non randomized MCT + physical activity	AD and Parkinson's disease	20	8	Cognition

*AD, Alzheimer's disease; CD, control diet; KB, ketone bodies; KD, ketogenic diet; KS, ketone supplementation; NCT, National Clinical Trial; PET, positon emission tomography; RCT, randomized controlled trial*.

## Discussion

Developing nutritional strategies to maintain cognitive abilities or improve quality of life of patients with AD or other neurodegenerative disorders has always been a research priority (1, 3). However, numerous nutritional interventions have failed to demonstrate significant effects on cognition (29–31). The Mediterranean diet represents an interesting preventive intervention but its efficacy is limited once diagnosed with AD (32). Saturated fat intake, which typically increases on KD, may also increase AD risk. In the Chicago Health and Aging Project, intake of high saturated fat was associated to a two- to three-fold increased risk of incident AD (33). However, 10 years later, the same authors acknowledged that the epidemiologic literature was seemingly inconsistent on this topic. For example, in the latter study, a decreased risk of AD was also related to consumption of monounsaturated and polyunsaturated fatty acids (34). Yet, high fat KD as well as MCT supplementation may positively influence the course of the disease, exerting a role on clinical symptoms (memory loss, executive function) but also on the pathophysiology of AD, in particular on inflammation or brain amyloid deposition (35).

During ketogenesis, the plasma concentration of KB (mainly β-hydroxybutyrate) gradually increases, and over 4 mmol/L, they become a source of energy for the central nervous system. In physiological ketosis, the concentration of KB does not exceed 8 mmol/L, blood pH remains stable, and glycemia, although decreasing, remains at its physiological concentration (36). Thus, KB can offset the absence of glucose act as an alternative fuel for the brain (37). In this context, KS or KD represent promising nutritional strategies to postpone cognitive decline due to AD, or even to *rescue brain energy*. As explained by Cunnane et al. while brain glucose uptake is compromised in MCI, brain ketone uptake and metabolism remain normal, in both MCI and mild-moderate AD (38). β-hydroxybutyrate and acetoacetate also demonstrated numerous benefic effects in neurological conditions, such as inhibit glutamatergic excitatory transmission in epilepsy and may also mitigate neuronal hyperexcitability resulting from AD neuropathology. The impact of these strategies even though symptomatic could be substantial given the number of people affected by AD. Besides, delaying the onset of behavioral symptoms or disability due to AD would lead to significant economic, public health and societal benefits (1).

Another original interest of the KD could be a reduction of carbohydrates consumption, which could trigger the brain amyloid accumulation. High glycemic diet was recently shown to influence change in amyloid levels, over the course of 1 year (6). In all participants, the association between change in amyloid retention and high glycemic diet was apparent in the precuneus. In prodromal AD, this brain area is specifically vulnerable to structural and functional alterations. Nevertheless, in participants with the highest amyloid status, this relationship extended into the posterior cingulate and lateral temporal lobe. These brain regions are highly susceptible to amyloid accumulation and metabolic impairment due to AD-related pathology. Thus, as demonstrated in animal models of AD, KD could play a direct protective role against the amyloid lesions of the disease (11).

However, an intriguing effect of KS is the cognitive improvement in healthy adults as well as in cognitively impaired patients, whereas the carbohydrates consumption remains equal. This effect could not be explained by the sole increase of energy supply to the brain, due to the rapid availability of KB following ingestion of MCT, since the doses used by several authors was low (22). The authors explained this gain in cognitive performance because of an increased rate of mitochondrial biogenesis. This phenomenon could be the benefic consequence of increased activation of PPARγ by KB.

Intermittent fasting has already been suggested as a therapeutic option for reducing the onset of neurodegenerative disorders (39). As in the KD, fasting induces a metabolic shift where energy results for KB. However, weight loss due to fasting is likely to accelerate cognitive decline, whereas maintaining stable weight and nutritional status are mandatory for patients with AD (23). Despite sufficient calorie intake, extreme carbohydrate restriction due to KD can profoundly affect diet quality. Low-carbohydrate diets are often low in thiamin, folates, vitamin A, vitamin E, vitamin B6, calcium, magnesium, iron or vitamin K. KD are typically low in fiber (40). In a recent prospective study, infants with drug-resistant epilepsy, who where started on KD (mostly 3:1), the incidence of adverse effects after one month of treatment was very high (91.7%), although none of them discontinued the diet for this reason (41). At diet initiation, the most common side effects were vomiting and hypoglycemia. After 1 month, the children mostly suffered from metabolic acidosis, constipation or vomiting. Non-compliance leading to diet interruption before 2 years of follow-up was reported in 21% of the infants. These findings confirm the long-term feasibility of such diet, in children. To determine whether similar results will be obtained with adults or older adults with AD remains a major challenge. In all cases, the monitoring of all adverse effects as well as the adherence to diet will be mandatory, in future longitudinal trials. Providing sufficient calorie and micronutrients is crucial for preventing the adverse consequences of nutrient deficiency. Therefore, an adequate long-term KD should also include appropriate vitamin supplementation. Finally, Bostock et al. described the potential adverse effects of self-administered KD from users of online forums: increased LDL-cholesterol levels, headache, fatigue, nausea, dizziness, digestive symptoms, and heart palpitations (42). Longer-term effects may lead to anemia, low bone mineral density, nephrolithiasis or cardiomyopathy (36).

## Conclusion

KD or MCT supplementation may represent promising options to fight against the cognitive symptoms of AD, especially in the prodromal stage of the disease. A growing body of evidence has suggested that KD could enhance cognitive performance and hopefully postpone cognitive decline in AD. Large sampled randomized controlled trials with long-term follow-up and appropriate clinical and biological outcomes (such as CSF or brain metabolism biomarkers) are needed to address the issues pointed out in this review. Special attention should be given to the onset of adverse effects (malnutrition and weight loss, digestive symptoms) and to the adherence to the diet, especially if these interventions are considered in the long term, as for the treatment of pharmacoresistant epilepsy. Whether cognitive gains would be maintained upon discontinuation of the KD (or KS) remains unknown so far.

The kind of KD suiting best for AD deserves further investigation: very restrictive diets may allow faster ketosis, but increase the risk of poor adherence. Recently, the Mediterranean diet has also been adapted to adhere to KD guidelines, and could represent a promising option to deal with the symptoms of neurodegenerative disorders.

## Author Contributions

ML and FM-L performed a review of the literature, analyzed and interpreted the data and drafted the manuscript. CP, MSO, and ED critically reviewed the first draft and revised the manuscript accordingly. All authors contributed to the article and approved the final submitted version.

## Conflict of Interest

The authors declare that the research was conducted in the absence of any commercial or financial relationships that could be construed as a potential conflict of interest.

## Publisher's Note

All claims expressed in this article are solely those of the authors and do not necessarily represent those of their affiliated organizations, or those of the publisher, the editors and the reviewers. Any product that may be evaluated in this article, or claim that may be made by its manufacturer, is not guaranteed or endorsed by the publisher.
